# Performance-Related Characterization of Forming-Induced Initial Damage in 16MnCrS5 Steel under a Torsional Forward-Reverse Loading Path at LCF Regime

**DOI:** 10.3390/ma13112463

**Published:** 2020-05-28

**Authors:** Kerstin Moehring, Frank Walther

**Affiliations:** Department of Materials Test Engineering (WPT), TU Dortmund University, Baroper Str. 303, D-44227 Dortmund, Germany; frank.walther@tu-dortmund.de

**Keywords:** torsional load paths, forming-induced initial damage, case hardening steel 16MnCrS5 (DIN 1.7139, AISI/SAE 5115), low cycle fatigue performance

## Abstract

Forming technology and in particular cold forward rod extrusion is one of the key manufacturing technologies with regard to the production of shafts. The selection of process parameters determines the global and local material properties. This particularly implies forming-induced initial damage in representation of pores. On this background, this study aims on describing the influence of these pores in the performance of the material 16MnCrS5 (DIN 1.7139, AISI/SAE 5115) under a torsional load path in the low cycle fatigue regime, which is highly relevant for shafts under operation conditions. For this purpose, the method of cyclic forward-reverse torsional testing was applied. Additionally, intermittent testing method and the characterization of the state of crack growth using selective electron microscopy analysis of the surface were combined. A first attempt was made to describe the influence of forming-induced initial damage on the fatigue performance and the crack growth mechanisms. The correlation of fatigue performance and initial damage was contiguous in the sense that the initial damage corresponds with a decrease of material performance. It was concluded that the focus of further investigations must be on small crack growth and the related material changes to identify the role of initial damage under cyclic loads.

## 1. Introduction

Shafts guarantee functionality in many technical applications like engines, powertrains or generators. These components are subtracted to multiple, in particular torsional loads in repeating cyclic sequences under operation conditions. The loads can thereby vary throughout lifetime with regard to the amplitude in dependence on the application. In the case of shafts, very high amplitudes can occur during especially starting of the loading phase. To guarantee the functionality and safety of these shafts, material tests under cyclic torsional load paths are necessary. The investigation of the material behavior under these operation conditions is of high relevance and prevails important information for the component design. As a matter of the design process, the material selection is of major significance. This is particular true, because the selected manufacturing technology, like forming for shafts, determines the material properties significantly as a matter of stress state during processing. The hardening degree, residual stress state or, in particular, the degree of forming-induced damage are the results of the selected process parameters. As for shafts, the technology of forming, viz. cold forward rod extrusion, gives broad possibilities to control the material properties globally and locally. This prevails the possibility of an operation load adapted component and material design. This is particularly true, due to the market share of cold rod extrusion of 21.5% on massive forming production, going ahead with 430 million produced parts in 2017 [1]. Thus, the understanding of interdependencies of material properties and operation behavior is of major importance. The importance thereby arises from the possibility to select the process parameter under consideration of the criteria of design and functionality, lifetime and operation load adaption. The present study addresses the characterization of the influence of forming parameter selection induced initial damage on the fatigue performance under cyclic torsional load paths in the regime of low cycle fatigue (LCF), viz. for high load amplitudes. The microscale dimension of initial damage is a main challenge, wherefore existing macroscale fatigue testing methods are evaluated on their usage potential.

Pre-deformation induced by load sequences either under operation or processing conditions, is known to influence the material properties and thereby the fatigue performance significantly [2]. This has in particular been shown for materials processed via the forming technology of forward rod extrusion for cyclic uniaxial operation conditions already in the 1985 [2]. Excluding forming-induced hardening and residual stresses on basis of simulation and experimental investigations [3], Tekkaya et al. have identified forming-induced initial damage in manifestation as pores as one of the determinants of material properties only recently [4]. The selection of the process parameters of the should opening angle 2*α* and the extrusion ratio *ε_ex_* was shown to determine the stress state during forming and thereby the degree of forming-induced damage as a function of triaxiality [3,5] points for cold forming bulk processes out that the cracking of non-metallic inclusions, like manganese sulfides (MnS), under states of high positive triaxiality induces local stress concentrations, which result in interface decohesion of the metallic matrix and the particular inclusion, especially at the tip of the inclusion. The latter can also occur without cracking of the inclusions [5] and is the second nucleation mechanism of initial damage. Once nucleated, the initial damage can go through the phases of growth and coalescence up to the occurrence of Chevron cracks [6].

The size (compare [7,8]), localization and orientation of pores in general are well known features that influence the behavior of casted [9] as well as forged [9] or additive manufactured [10] materials and induce anisotropic material behavior under cyclic loads. The main focus of the studies performed is on uniaxial testing conditions, whereby torsional load paths were investigated less common and mainly in recent years. Reference [9] showed for AZ31B MG alloy the increase of fatigue life time up to failure with reduction of porosity. Reference [10] pronounced the fatigue life determining effect surface defects for additive manufactured Ti-6Al-4V alloy and related the crack propagation direction to the weakest plane according to the build direction, additionally highlighting the reduced shear ductility. Moreover, the relevance of small crack growth was discussed in connection with initial surface pores [11].

Focusing on the mechanism, Endo et al. have shown, for the heat-treated steel C35 with banded and not-bended ferrite-pearlite microstructure that a macroscopic shear mode failure went ahead with a continuous Mode I small fatigue crack growth under torsional load paths [12]. Endo et al. found that the final failure was induced by crack branching on the surface, whereby the crack propagated firstly in the ferrite phase under pure torsional Mode II conditions and secondly under Mode I conditions in a mixed structure of ferrite and pearlite grains up to fracture. It was concluded that a threshold level for Mode I crack growth existed that was to overcome. The contribution of internal defects with microscale sizes and non-metallic inclusions was not addressed. Studies with similar findings for comparable microstructures were performed by Pokluda et al. for long fatigue shear mode cracks [13,14] focused on the crack branching phenomena, finding that a decrease in shear stress results in a decrease in longitudinal crack length.

The correlation of fatigue performance and defects has been addressed by multiple authors like Murakami [15,16] or Beretta [17]. The limitation of these studies was the consideration of cracks sized at least *l_min_* = 40 µm, whereby different sources of crack or pore initiation like the manufacturing process, resulting in complex and heterogeneous crack geometries, pre-deformation or idents have been investigated. The limitation of fatigue performance was pre-dominantly traced back to crack growth threshold relations. Murakami et al. have shown that defects determine the fatigue performance of materials by the threshold condition for small crack nucleation at the tip of the defects [15]. Later studies conducted on specimen containing holes of *d_ini_* = 40 µm and being pre-cracked up to an initial crack length of minimum *l_min_* = 200 µm under cyclic tension-compression load paths, showed the correlation of the torsional fatigue limit and the threshold for non-propagating branched cracks [16]. The effect of artificially induced cracks at a length scale of more than 40 µm on the crack growth threshold under torsional loading paths was addressed by Beretta et al. for SAE 5135 gear steel, finding that the value of stress concentration parameter significantly influences the crack propagation mode [17]. Studies addressing the influence of defects on length scales down to *l_min_* = 5 µm are to the best knowledge of the editor not available.

From this background, the need arises to address fatigue performance under torsional loadings in dependence on defects of a microscale length far below the sizes investigated in recent studies. The current study addresses this need while characterizing the fatigue performance for a forward-reverse torsional loading path firstly on a macroscale (Section 3.1). A constant rotation amplitude was selected in order to address the lower regime of low cycle fatigue (LCF). Additional intermittent tests were performed in combination with meso- and microscopic analyses for correlation and identification of the influence of forming-induced initial damage (Section 3.2). Therefore, the analyses focused predominantly on crack growth mechanisms and interactions on the free surface, and to a minor degree on microstructural phenomena. In the end, a first attempt was undertaken to address the basic requirements for further investigations.

## 2. Materials and Methods

### 2.1. Material

The experimental data presented base on the case hardening steel 16MnCrS5 (DIN 1.7139, AISI/SAE 5115), which was provided by the material supplier Georgsmarienhuette. The material was delivered in a rolled and drawn, ferrite-pearlite annealed state (+FP) in bars with diameter *d_ini_* = 40 mm and length *l_ini_* = 750 mm. The general chemical composition is listed in Table 1. The maximum tensile strength was *R_m_* = 715 ± 12.5 MPa.

**Figure 1:**
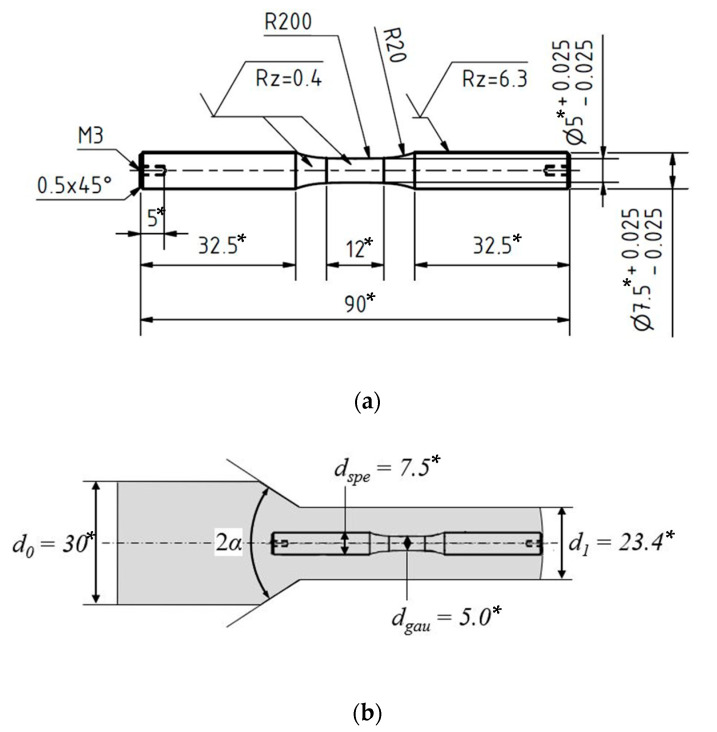
(**a**) Specimen for torsional fatigue testing; (**b**) Schematic visualization of extraction position. * All dimensions are in mm.

The initial material was manufactured to cylindric workpieces, billets, with diameter *d_priorExtr_* = 30 mm and length *l_priorExtr_* = 71 mm. These cylindric work pieces were extruded to a diameter of *d_Extr_* = 23.4 mm (see Figure 1b), corresponding to a forming degree of 0.5. Two different extruded material states E.530 and E.590 were investigated. The state E.530 was cold forward rod extruded with the process parameters 2*α* = 30° as shoulder opening angle and *ε_ex_* = 0.5 as extrusion strain, whereas the state E.590 was processed with *2α* = 90°. Due to the increase of the shoulder opening angle, the stress state during forming increased. The higher stress state during extrusion of the material state E.590 resulted in a higher degree of initial damage compared to the material state E.530. The state of initial damage is detailed in Section 2.3.1. For the extrusion process a hydraulic triple action press (HZPUI, SMG, Mannheim, Germany) with a punch speed of *v_punch_* = 10 mm/s was used [19].

### 2.2. Material State and Characterization Methods

The material state was characterized applying the methods listed in Table 2. The initial as well as the material states after distinct cyclic loading cycles has been characterized using the methodology of scanning electron microscopy (SEM) in combination with secondary electron (SE) analysis, energy dispersive x-ray spectroscopy (EDS) and electron backscatter diffraction (EBSD). The SEM analysis were performed using a scanning electron microscope (Mira 3 XMU, Tescan, Brno, Czech Republic), equipped with an EDS-detector (Octane Pro, EDAX/Ametek, Berwyn, PA, USA) respectively an EBSD-detector (DigiView 5, EDAX/Ametek, Berwyn, PA, USA) for EDS and EBSD analyses, respectively. The quantification of the damage state with regard to the absolute area of pores has been conducted using a grey value and EDS-based particle counting methodology (PCM). The PCM analyses were carried out using the commercial PCM genesis software (EDAX Particle Analysis, Genesis 3.2, EDAX/Ametek, Berwyn, PA, USA) in the way stated in [3]. The SEM analyses were conducted on cross sections after grinding from P320 down to P2500 grade and polishing with diamond suspensions of 6 µm, 3 µm and 1 µm. The surface was finished polishing with an OPS suspension.

The analyses of the residual stress state (RSA) have been conducted using x-ray diffractometry (XRD, Bruker). The residual stress states were analyzed at the surface of the tested specimens by using a x-ray diffractometer (D8 Discover, Bruker, Billerica, MA, USA) with a copper X-ray tube in side inclination and Bragg-Brentano geometry with sin²ψ method.

The hardness tests (HT) according to Vickers (HV) have been carried out with a testing force of 98.07 N (HV10). The device used in this study was the Shimadzu HMVG hardness testing system (HMV-G, Shimadzu, Kyoto, Japan) [20].

### 2.3. Methods for Characterization of Fatigue Behavior

#### 2.3.1. Sample Geometry, Material and Surface Conditions

The specimens were machined out of the bar material respectively the cold forward rod extruded workpieces via turning in axial (longitudinal, *l*) direction. The surface of the specimen was grinded from P320 down to P2500 grade and finally polished with diamond suspensions with particle sizes from 6 µm, 3 µm to 1 µm. The sample geometry used for the investigations is shown in Figure 1a, the corresponding area of extraction out of the workpiece is depicted in Figure 1b. The area represents according to [3] the area of the workpiece within which the stress state during cold forward rod extrusion is homogeneous with regard to the forming induced initial damage and the hardness.

**Figure 2:**
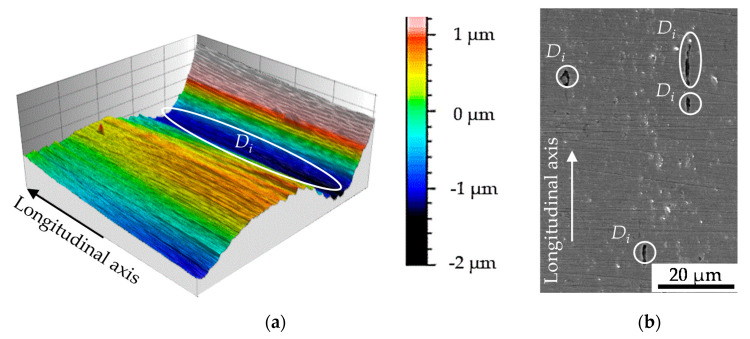
(**a**) Surface profile of the specimens; indicating a exemplary depth of initial damage (*D_i_*) of up to 2 µm; (**b**) Forming-induced initial damage (*D_i_*) at the specimen surface after manufacturing.

The obtained surface roughness was *R_a_* = 0.327 µm as detected by the confocal white light interferometer (µsurf, NanoFocus, Oberhausen, Germany). A section of the resulting roughness profile is shown in Figure 2a. The influence of the initial damage at the surface according to Figure 2b can be seen in the depth profile provided. Being manufactured out of the work pieces and due to the high requirements on the surface quality of minimum *R_z_* = 0.4 µm obtained by polishing, the damage state at the surface is to some extend comparable with the initial damage in the material volume. Limitation arise with regard to the decohesion of non-metallic MnS inclusions present in the micro-alloyed case hardening steel. The initial damage in the volume manifested pre-dominantly as interface decohesion of the metallic-matrix and the MnS non-metallic inclusion or metallic matrix decohesion at the tip of the MnS inclusion, grown isotropic in direction of extrusion, viz. fiber direction, during cold forward rod extrusion (see [19]). The difference in initial damage was quantified by means of the area of initial damage by [19] with ∆*A_in, d_* = 218 µm² (*A_in, d_* = 84 µm² for the material E.530 and *A_in, d_* = 302 µm² for material state E.590).

The Vickers hardness level (HV10) was with 230HV10 comparable for both material states. The residual stress state at the surface of the specimen characterized by x-ray diffraction analyses, was negligible. The grain size was for both materials in the range of 9.2 ± 0.2 µm in longitudinal and 6.5 ± 0.3 µm in transversal direction. Both material states were textured in extrusion direction <100> with regard to the specimen longitudinal axis.

#### 2.3.2. Testing Set-up and Methods

**Figure 3:**
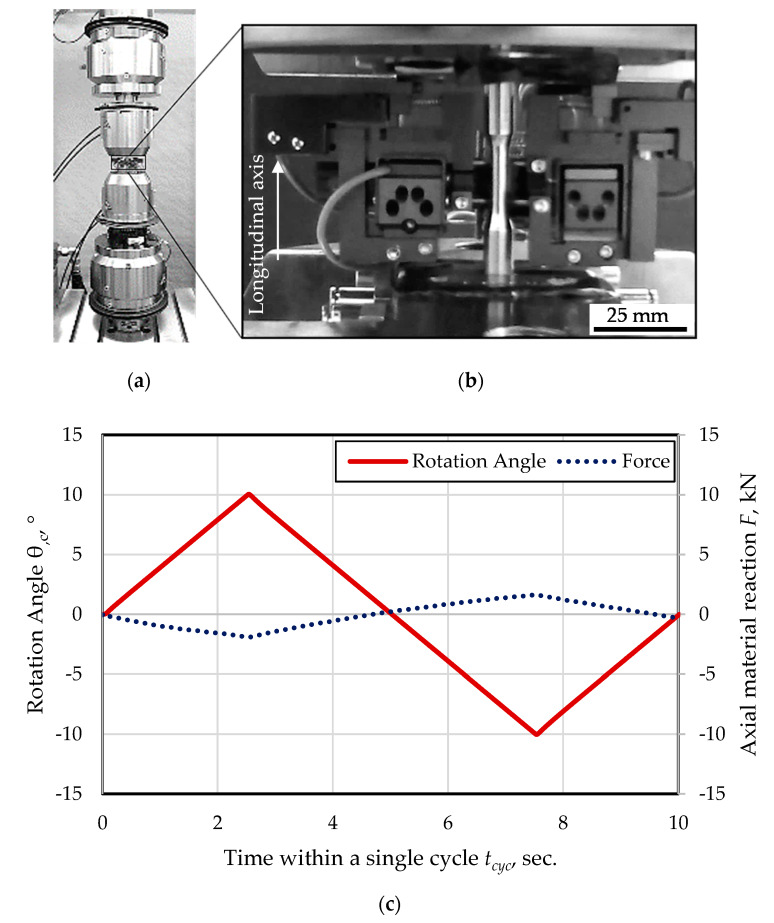
(**a**) Testing system Walter+Bai LFV-T250 T2500 HH; (**b**) testing set-up with specimen, axial-torsional extensometer type Epsilon^®^ Tech 3550-010M-020-004-ST with gauge length *l_gau_* = 10 mm used for cyclic forward-reverse torsion tests with fixed specimen length; (**c**) Load path in rotation control and resulting axial material reaction.

The experimental set-up is depicted in Figure 3a. The fatigue tests were performed using the axial-torsional testing system (Walter+Bai, LFV-T250 T2500 HH, Löhningen, Switzerland) with a maximal nominal axial load *F_nom_* = 50 kN and a maximal torsional load *M_t,nom_* = 100 Nm. To detect the axial and torsional deformation during testing, a tactile, axial-torsional-extensometer (Epsilon^®^ technology, Epsilon^®^ Tech 3550-010M-020-004-ST, Jackson, Mississippi, USA), as shown in Figure 3b was used. The gauge length was *l_gau_* = 10 mm in axial direction. The testing frequency was *f_tors_* = 0.1 Hz. The tests were conducted in torsional angle control with a ratio of *R_tors_* = −1. The forward-reverse load sequences were applied in form of triangular endurance load cycles. The tests were conducted as constant amplitude tests (CAT) at a rotation angle amplitude of *θ_c,a_* = 10°. The length change of the specimen due to drilling was compensated. The load amplitude was selected in order to address the upper low cycle fatigue regime for identification of relevant mechanisms. The load path is depicted in Figure 3c. The run-out lifetime was set to 1000 cycles. The lifetime tests were conducted continuously, whereby intermittent tests were additionally performed in order to characterize the cyclic damage evolution on the surface of the specimen. The current number of cycles at the evaluated state within the lifecycle is indicated with *N_i_*. While applying this intermittent testing method (ITM), the constant amplitude tests were interrupted at distinct numbers of cycles, i.e., fatigue (damage) states.

## 3. Results

### 3.1. Fatigue Performance under Forward-Reverse Torsion Conditions with Position Control

**Figure 4:**
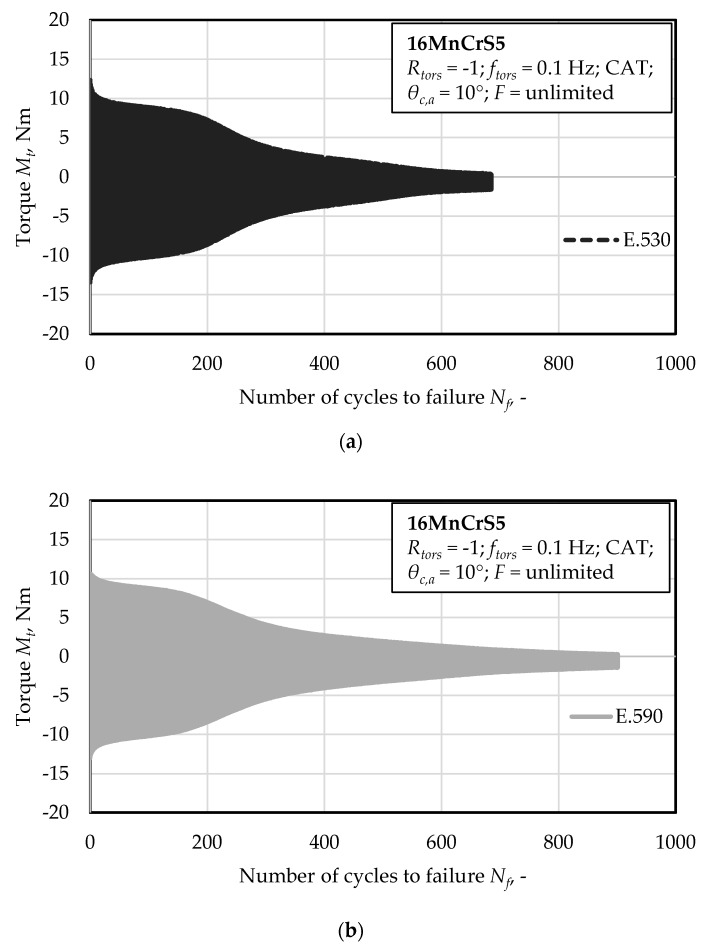

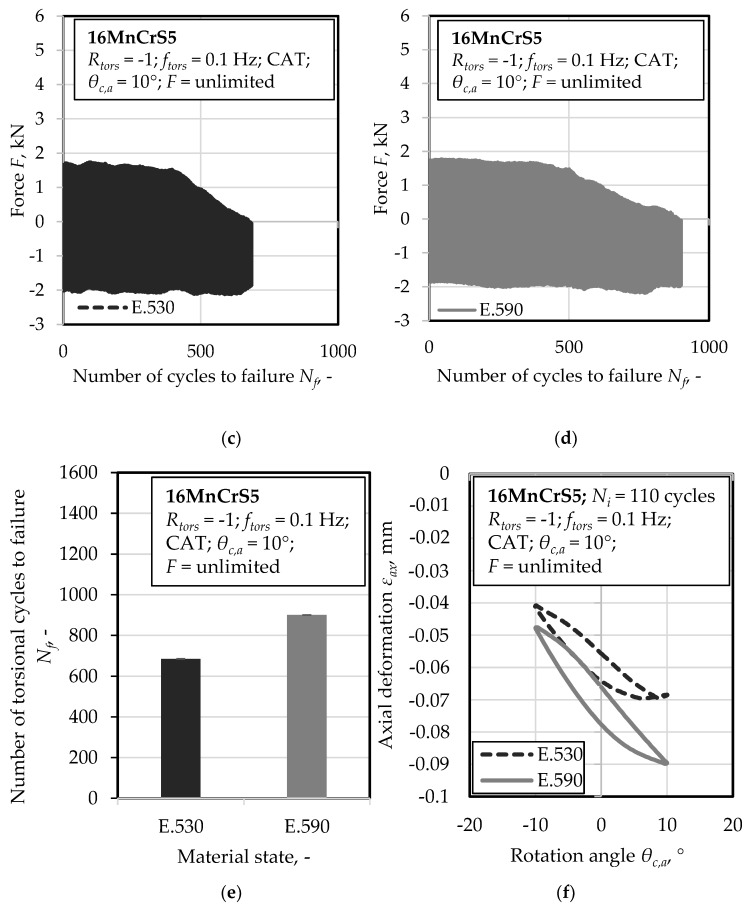
Fatigue performance for material states E.530 and E.590 in rotation angle control under torsional, constant amplitude load paths with regard to torque level for material state (**a**) E.530; (**b**) E.590; force level for material state (**c**) E.530; (**d**) E.590; (**e**) torsional cycles to failure; (**f**) axial deformation *ε_ax_* after an indicated number of torsional cycles during the ongoing lifecycle *N_i_* = 110 torsional cycles.

The fatigue tests performed under cyclic torsion testing conditions at a constant rotation angle of *θ_c,a_* = 10° with axial position control, showed a correlation with countervailing tendencies between fatigue performance and degree of initial damage for the material states E.530 and E.590, respectively. As can be seen in Figure 4a,b, the torsional load induced under forward-reverse torsional fatigue in angle control, the resulting torque moment induced in the material state E.530 was higher compared to material state E.590. The material E.530 with the lower initial state of damage showed a worse performance with regard to the number of cycles to failure as depicted in Figure 4e. This decrease of performance existed from the beginning of the loading sequence in manifestation of a reduced load bearing capacity in terms of maximum torque level arising in the material (Figure 4a,b). Material state E.530 shows an increased speed and reduced time of cyclic damage evolution. The resulting force level is shown in Figure 4c,d for material state E.530 and E.590, respectively. The comparison of the torque and force level indicates and comparable small degree of tensile, compression force of approximately ±2 kN. Therefore, a compression-tension anisotropy arose, which was stronger for the material state E.590. This went ahead with an increased compression stress state and a reduced tensile stress state for the material state E.530 during testing.

At the background of Figure 4a, the obtained higher torque level during testing correspondents with the decreased performance of material state E.530. This is particularly true, when referring to the number of torsional cycles to failure (Figure 4e). The axial-deformation/rotation angle hysteresis shown in Figure 4f, indicates an increased defor-mation of the material state E.590 in axial direction, which has the increased degree of forming-induced initial damage. Thus, the axial deformation corresponds positively with the degree of initial damage. More initial damage has induced a higher and easier deformability in axial direction. The resulting torque moment during testing overcompensates this effect.

This particularly was accompanied by a higher deformation of the specimen in axial direction, *ε_ax,_* (Figure 4f), which showed a decreasing tendency throughout the lifetime. A forward-reverse as well as compression-tension asymmetry for the corresponding half-cycles arose during testing (Figure 4a–d). Simultaneously, the resulting axial loads in the material were higher in tension for the material state E.590 compared to the material state E.530 and less high in compression. Despite the higher tensile stress state, the material state E.590 performed better under torsional loading paths.

### 3.2. Crack Path Analysis/Identification of Mechanisms

#### 3.2.1. General Cracking Mechanisms (Surface and Volume)

**Figure 5:**
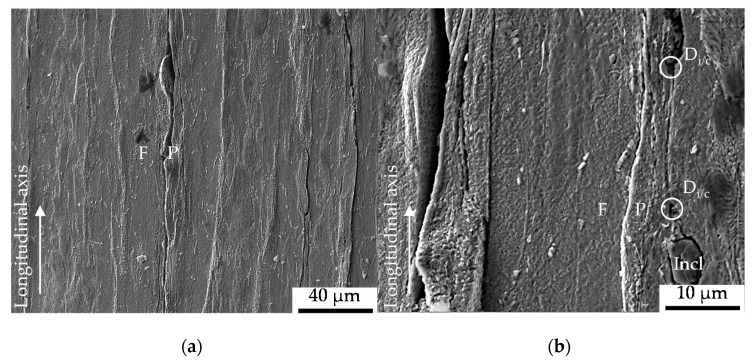
Cracks grown at the surface in axial direction after intermittent fatigue testing and surface etching with Nital 3% (**a**) overview indicating the crack localization within the ferrite (F) – pearlite (P) microstructure; (**b**) non-metallic inclusions (Incl) as part of the crack path and inclusion/matrix-decohesion assisted isotropic pore growth.

To characterize the crack growth mechanisms for understanding the macroscopic material behavior, the intermittent testing method was used. A distinct interruption point after 100 cycles was investigated. For the applied load amplitudes resulting in low cycle fatigue lifetime behavior, the nucleation of cracks in axial direction (see Figure 5a,b) occurred in the very first cycles of the fatigue life.

As typically for LCF-crack growth behavior, the phase of crack nucleation was thereby found to have a minor partition on the whole life cycle. Nevertheless, this phase was determinant for the cyclic damage evolution and, thus, the fatigue performance of the tested specimen. This is particularly due to the significant dependence on the surface conditions as reference tests with deliberately changed polishing direction have shown.

**Figure 6:**
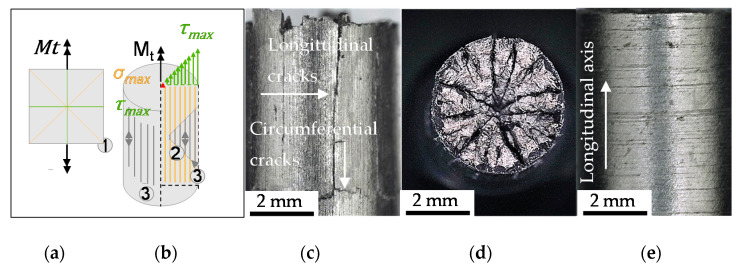
(**a**) General principle schema of the directions of maximal principle stress *σ_max_* and maximal shear stress *τ_max_* under pure torsional load paths; (**b**) General principle schema of cyclic damage evolution under torsional load paths with the applied torque moment M_t_; (**c**) longitudinal cracks in main shear direction and crack coalescence at the circumference; (**d**) crack propagation paths in radial direction; (**e**) divergent failure mechanism induced by cracks in direction of the circumference.

According to Figure 6e, the direction of remaining polishing remarks induced the direction dependent crack nucleation. Despite no other indication, the secondary influence of the ferrite-pearlite banded structure was not found to be excluded from the performed investigations. Figure 5a shows the ferrite-pearlite phase distribution at the surface after etching and indicates a ferrite-pearlite interface as well as pearlite localization of the longitudinal cracks (Figure 5b). Due to the statistically localization of initial damage at non-metallic inclusions, a contribution of initial damage was also not neglitable. This is particulary true, because the inclusions and therby the initial damge were found to be included in the crack paths via pore intgration, respectively isotropic crack growth and coalescence (see Figure 6c).

The sequence of crack propagation phases is schematically depicted in Figure 6b. Nucleating at the middle section of the tapered sample on the circumference, the cracks propagated in axial direction with degressive growth rate in dependence on the distance from the tapered section, viz. from the length of the longitudinal cracks and the number of cycles. The crack growth was shear stress controlled with crack opening Mode II in direction of maximal shear stress *τ_max_* (see Figure 6a). After sufficient decease of crack growth, the longitudinal crack branches in circumferential direction (see Figure 6c). The further crack propagation was investigated to proceed in radial direction, principle stress driven *σ_max_* (see Figure 6a) in an angle of 45° (Figure 6d) up to final fracture of the specimen.

#### 3.2.2. Mesostructural Correlation (Surface)

**Figure 7:**
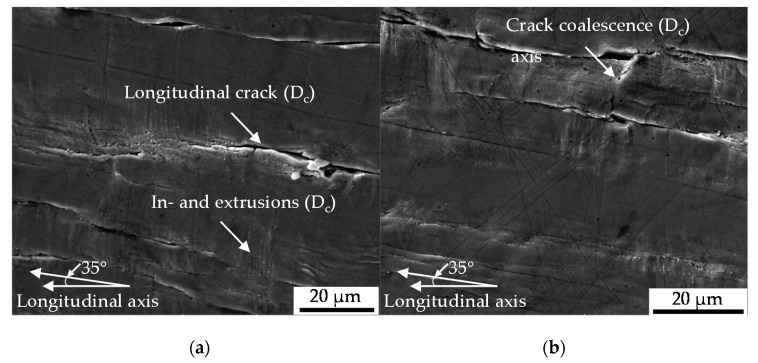

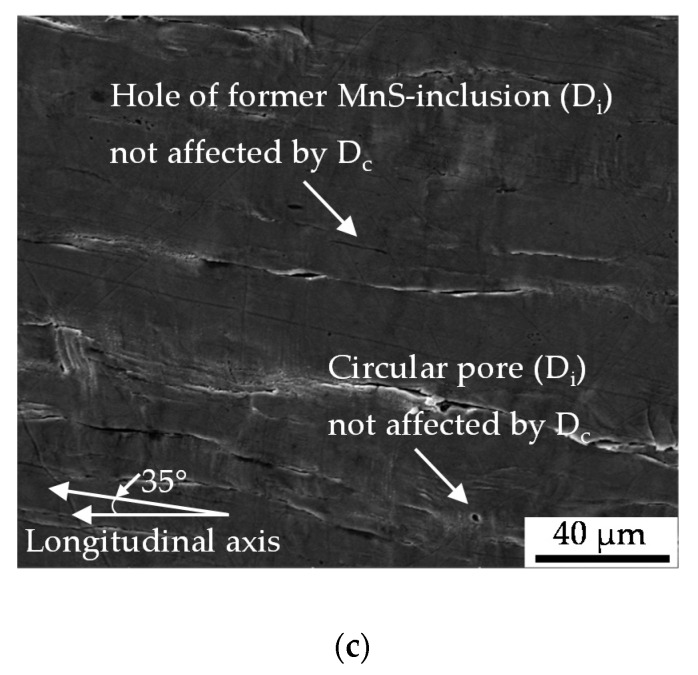
Stages of cyclic damage (*D_c_*) evolution (**a**) early stages; (**b**) advanced stages; (**c**) with initial forming induced damage (*D_i_*).

**Figure 8:**
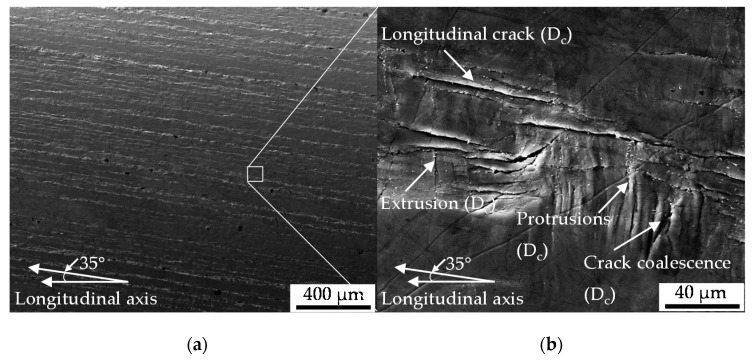
(**a**) Overview of sample surface after cyclic torsional load path with longitudinal cracks evolving at sample surface in axial direction; (**b**) interacting different types of cyclic damage (*D_c_*).

The results were obtained characterizing the crack growth at the surface microscopically. Therefore, the testes were interrupted in the intermittent testing methodology after a distinct level of 100 cycles. Nucleation and growth of the longitudinal cracks was accompanied by the development of in- and extrusions Figure 7a and growth in direction of the circumference (Figure 7b up to crack nucleation assisted by the protrusions (Figure 8b) and crack coalescence (Figure 7b and Figure 8b). The contribution of initial damage was found to be heterogeneous, dependent on the type of initial damage. Initial damage at the tip of MnS inclusions tended to merge to the longitudinal cracks, so that the contribution was detectable in the early stages of damage evolution (Figure 7a). The contribution was merely indirectly traceable with proceeding cyclic damage evolution due to interaction and superposition with cyclic damage (see Figure 8b).

Therefore, exceptions occurred, accompanied by missing shear bands, viz. missing localized slip, and consequently missing protrusions in close distance (see Figure 7c). This finding was approved for initial damage in manifestation of circular pores (see Figure 7a). The presented mechanisms were not found to vary for the investigated high amplitudes leading to several cycles to failure of not more than 1000 cycles and thus the lower part of the of low cycle fatigue (LCF) regime.

#### 3.2.3. Mesostructural Correlation (Volume)

**Figure 9:**
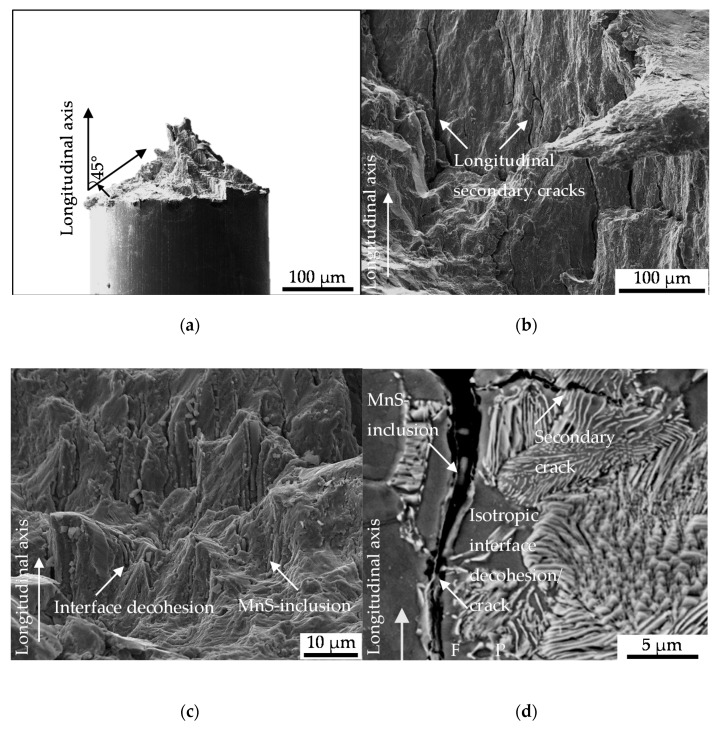
(**a**) Fractographic macro crack paths; (**b**) longitudinal cracks evolving in the material volume; (**c**) interface decohesion of metallic matrix and MnS inclusion due to cyclic damage evolution; (**d**) exemplary ferrite (F) – pearlite (P) phase localization of longitudinal cracks following isotropic crack growth in the material volume visualized after performed etching with Nital 3%.

The results were obtained by fractographic SEM-analyses of the fatigued specimen after failure. The cyclic damage evolution proceeded into the volume at approximately 45° in direction of maximal normal stress up to final fracture of the specimen (Figure 9). The material degradation was assisted by the evolution of cracks in the volume in longitudinal direction. Therefore, cracks nucleated at MnS inclusions, viz. the preferred localization of initial damage, were shown to have a central role (Figure 9c,d). This was found for the final fracture as well as for the ductile fracture.

**Figure 10:**
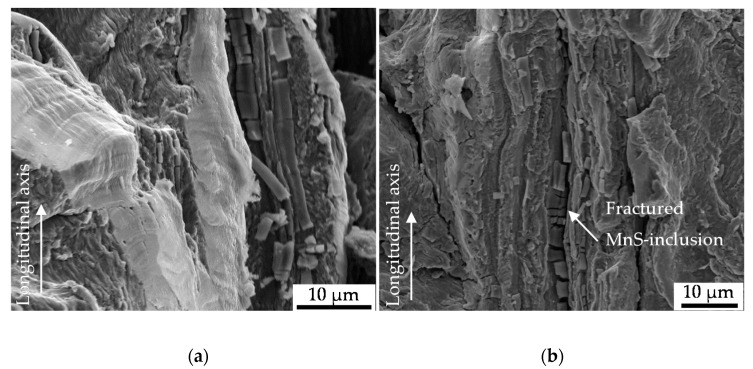
(**a**) Segments of cyclic damage evolution; (**b**) MnS-inclusions fractured due to cyclic damage evolution under torsional load paths.

The cyclic damage evolution under the investigated torsional load path evolved basing on different mechanism, as indicated by segments of different fracture morphology and particularly, differently pronounced striations (Figure 10a). Irrespective of the degree of ductility of the cyclic damage evolution, indicated by the striations, no direct indicator of initial damage was detected. Secondary cracks could not have been related to initial damage. Tire tracks merely indicated the contribution of detached particles or protrusions to cyclic damage evolution and emphasized the lateral damage evolution under torsional load paths (Figure 10b). No correlation with initial damage was scientifically indicated.

#### 3.2.4. Microstructural Correlation (Volume)

**Figure 11:**
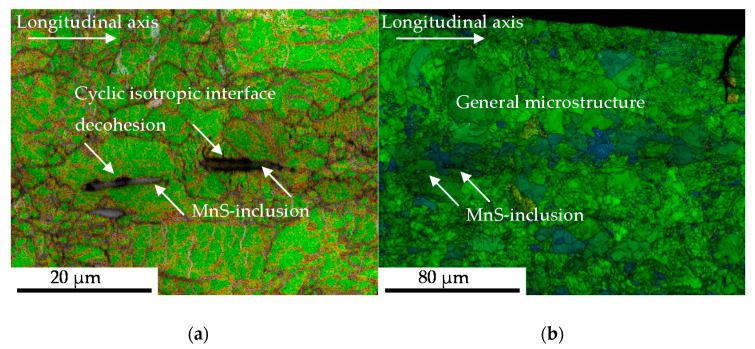

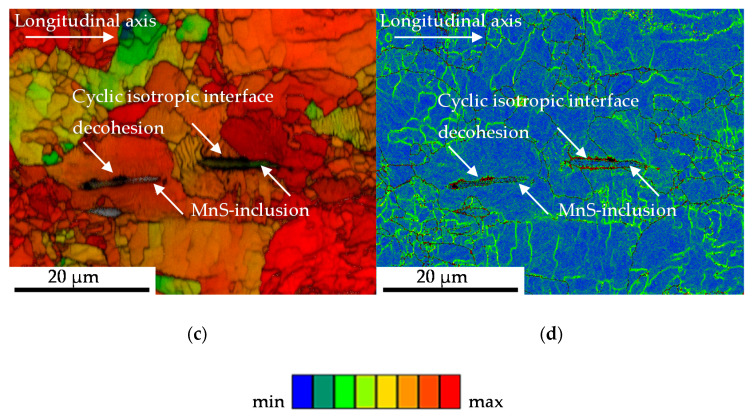
Electron Backscatter Diffraction-based analysis of MnS-inclusions and forming induced initial, respectively cyclic damage, in dependence on the microstructure after torsional loading with (**a**) Geometric necessary dislocation (GND) map; (**b**) Kernal average misorientation (KAM) map in higher magnification to give an overview over the surrounding microstructure; (**c**) Schmid-parameter map; (**d**) Grain average misorientation (GAM).

For microstructural correlation cross sections of the failed specimen were prepared and characterized using EBSD-methodology in the SEM. The torsional load path induced a change in preferred orientation from <100>-direction, viz. longitudinal direction, in <111>- and <001>-direction indicated by EBSD measurement and analysis of the texture. Facing on MnS inclusions the material state after intermittent testing after *N_i_/N_f_* = 100%, Pores in longitudinal direction where considered to be initial damage, due to the circumstance that the maximal tensile force evolving during testing due to twisting of the sample was *F_max_* = 2.5 kN. As depicted in Figure 11, an interface decohesion of the metallic matrix and the MnS inclusions following the “ideal” direction of maximal shear stress under torsional loads took place. This finding was not transferable to pores, considered to be initial damage, at the tip of the MnS inclusions orientated in longitudinal direction (compare Figure 11d). The initial damage was additionally located in grains, which had a comparable high Schmid-parameter with regard to tensile load paths (compare Figure 11c), showing little density of geometrical necessary dislocations and misorientation according to EBSD-based approximation (Figure 11).

## 4. Discussion

The results of the mechanical testing under cyclic forward-reverse torsional load paths obtained, indicate a complex contribution of initial damage on the cyclic performance.

It is concluded from the mechanism detected on the surface on the mechanism in the volume accompanied by the preparation of cross section. The latter has even under the most precise care while preparing some influence on the results obtained. As [21] pointed out, non-destructive methods of state characterization are firstly not available due to the micro scale dimension of the initial damage and secondly due to the density of the investigated steel. Frequently used devices like µ-computer tomography (µ-CT) are limited with regard to both. References [19] and [21] discussed and highlighted the advantages of the cross-section preparation.

The nucleation and growth of the non-propagating longitudinal cracks on the surface of the specimen (Figure 7 and Figure 8) is in well agreement with the findings of [12]. Endo et al. highlighted in particular the nucleation of Mode II cracks in areas with broad partition of ferrite grains like the area where the grains are orientated in longitudinal direction due to rolling or extrusion [12]. The crack path analysis conducted in this study varied in the sense that a contribution of initial damage localized in the interface of ferrite and pearlite grains (Figure 5a) or merely pearlite grains (Figure 5b) on the nucleation and growth of macrocracks in longitudinal direction was indicated. This was particularly due to the shown involvement of inclusions within the phase of longitudinal crack growth. Because these cracks were found to be non-propagating cracks in the end, a significant contribution of initial damage might be without significance. This surely implies the very first phase of crack nucleation on the surface, but under the assumption of comparable mechanism determining the crack growth mechanisms in the volume also the following phases up to final failure. The contribution of MnS inclusion cracking in the volume on the evolution of cyclic damage, viz. the degradation of the material, as is depicted in Figure 9b,c, in combination with the preferred localization of initial damage close to this inclusions, might be seen as an indication of mode of action. Against this background, the absence of secondary cracks in radial direction in the material volume is an argument in the sense that the contribution of initial damage is describable with the acceleration of isotropic crack growth in longitudinal direction. The fractographic morphology and especially the absence of striations (Figure 9b) the role of plasticity reduced mechanisms, but indicate no influence of initial damage. The influence of MnS inclusions in the sense of reducing ductility, is in agreement with the findings of [22] and [23]. The mechanism of cracking need to be questioned due to the findings of [24]. Kage et al. indicated for tension-compression and bending fatigue that loads applied in the longitudinal direction of the specimen promote fatigue crack nucleation from slip bands and grain boundaries, whereas cracks were found to nucleate at inclusions under loading in direction of the specimen thickness [24]. Transferred to the torsional testing procedure applied during this study which went ahead with a superposition of axial loads due to the compensated length change due to twisting, the influence of initial damage might be further reduced. The overlay with the influence of MnS inclusions might be significant. In particular, due to the high amplitudes going ahead with the investigated regime of Low cycle fatigue (LCF) and the torsional loading perpendicular to the extrusive flattened MnS inclusions as described by Temmel et al. [25]. The relatively high hardness of the extruded material investigated, might pronounce the role of the MnS inclusions additionally according to [26]. Due to the fact that the hardness was found to be comparable for the material states E.530 and E.590 (see Section 2.3.1), the influence of the MnS inclusions is comparable. The high contribution of these onto cyclic failure might overcompensate the differences in initial damage between the two material states investigated.

The crack coalescence in the pearlite phase shown in (Figure 5b) might be taken in consideration for the explanation of the elongated phase of decreasing maximum torque level of the material state E.590 compared to E.530 (Figure 4). The increased partition of initial damage, viz. pores and cracks, in the material state E.590 might evoke an increased degree of local stresses and thereby hardening due to non-propagating, blocked micro-cracks in the pearlite phase. The role of micro-mechanical fields on the crack propagation as discussed in [27] must additionally be taken into consideration [27]. This additional resistance to crack growth might contribute to the elongation of the material degradation in the sense of increased number of cycles for reducing the torque state in the material up to failure (Figure 4a,b). Since the pearlite phase is scientifically proved to be hard and to contribute to the cyclic failure only after the ferrite phase, no contradiction arises and is supported by the findings of Figure 11. The in Figure 11 shown interface decohesion of metallic-matrix and inclusion in radial direction indicates no enhanced contribution to material failure, despite the surrounding microstructure indicates more forming induced damage. The shown misorientations might not support that theory, which agrees with the findings of Gruenewald et al., who recently highlighted that is not suitable for detecting the crack growth resistance for stage-II fatigue cracks for cubic face centered. materials [28]. The detected not increased density of geometrical necessary dislocations (GND, Figure 11) might be not contractionary, due to the fact that the partition and contribution of statistical stored dislocations (SSD) might be enhanced due to the location of the MnS inclusions in the grains. Taking into consideration that the SSDs cannot be detected using EBSD, further investigations with the electron channeling contrast imaging (ECCI) method are necessary for confirmation of that theory.

The finding of longitudinal cracks nucleating and growing in the ferrite phase is in agreement with the findings of Endo et al. for ferrite-pearlite steel in a rolled condition [12], whereby the findings are extended by the role of initial surface damage on the nucleation and growth of the longitudinal cracks. The for the material state E.590 increased evoking tensile forces (Figure 4c,d) might be correlated with the increased pore partition and thereby, the reduced load bearing capacity in longitudinal direction. The role of the increased compression forces and the increased axial deformation might be discussed equivalently and furthermore, support the theory of initial damage induced increased crack propagation resistance. The reduced load bearing capacity of the material state E.530 in the first cycles cannot be explained basing on the results obtained during this study. There might also be some softening of the extruded material occurring, like discussed by [29] for torsional low cycle fatigue loads due to redistribution of plastic strains and strain transfer between ferritic and austenitic phases [29] occurring and interfering. The for the first cycles shown high plastic deformation indicated by the broad axial deformation/rotation hysteresis that was found to decrease with increasing number of cycles might indicate softening processes under the high loads in the first cycles and torsional hardening in the following cycles. Additional research is necessary with regard to this phenomenon.

The visualized cyclic torsional deformation mechanism indicates a dependency of the initial damage with the process of damage evolution. Therefore, the dependence of the influence on the phase orientation of the initial damage became obvious.

Further research activities should focus on the effect of cycling softening due to the fact that the investigations were carried out for on extruded and thereby hardened material that tends to undergo cyclic softening. In addition to this, the effect of hardening in the surrounding of initial damage and residual stresses must be addressed in order to solve the remaining questions and uncertainties. That might include in situ tests of primary small crack growth mechanism under relevant crack opening mode conditions, supported by successive characterization of the material state. A stress relief potential for MnS inclusions implying less cracking due to the stress concentration at the initial damage and non-propagating microcracks instead of the inclusions should be considered. Investigations are necessary to address the scientific question, whether there are interdependencies of crack branching and initial damage.

## 5. Conclusions

The present study has shown that forming-induced initial damage is not necessarily resulting in a decreased material performance under torsional loading paths. This was shown for load cases of cyclic forward-reverse torsional loading paths with fixed length of the specimen, viz. no degree of freedom for movements in axial direction. Additionally, a first attempt was undertaken to show the influence of initial damage on the material performance as well as the interdependencies of initial and cyclic deformation mechanism. The influence was indicated to manifest pre-dominantly in isotropic crack growth in longitudinal direction and thereby in direction of the ferritic-pearlite banded structure induced by cold forward rod extrusion. It was discussed that the influence might be overcompensated while accelerating the crack growth in longitudinal direction, which is only of secondary relevance for the propagation of failure inducing shown mode I crack growth in degree of 45° to the longitudinal axis, viz. in direction of the maximal principle stress. The relevance of further investigations in particular with focus on small crack growth and branching mechanisms was highlighted. There might arise broader freedom in terms of process parameter selection for the forming processes, as far as the influence and mechanism of initial damage are clarified.

## Figures and Tables

**Table 1:** Chemical composition of the investigated low alloyed case hardening steel 16MnCrS5, in wt% [18].

Material	C	Si	Mn	S	Cr
16MnCrS5	0.14–0.19	≤0.4	1.0–1.3	≤0.02–0.04	0.8–1.1

**Table 2:** Characterization methods and application [20].

	Characterization Method and (Device)					
Characterization Application	SEM (PCM)	SEM (SE)	SEM (EDS)	SEM (EBSD)	XRD (RSA)	HT (HV)
General microstructure	-	X	-	-	-	-
Grain size	-	-	-	X	-	-
Grain orientation	-	-	-	X	-	-
Damage state	X	-	X	-	-	-
Residual stress state	-	-	-	-	X	-
Initial hardness level	-	-	-	-	-	X
